# Supplementary material for: Similarity of introduced plant species to native ones facilitates naturalization, but differences enhance invasion success
Source: Nat Commun. 2018 Nov 6;9:4631. doi: 10.1038/s41467-018-06995-4 (PMC6219509; doi:10.1038/s41467-018-06995-4)
Supplement: Supplementary file 1 — Supplementary Information [file 41467_2018_6995_MOESM1_ESM.pdf]

SUPPLEMENTARY INFORMATION

Similarity of introduced plant species to native ones facilitates naturalization, but differences enhance invasion success

Divíšek et al.

Contents

Supplementary Figures....................2
Supplementary Tables....................19
Supplementary References....................28

Supplementary Figures

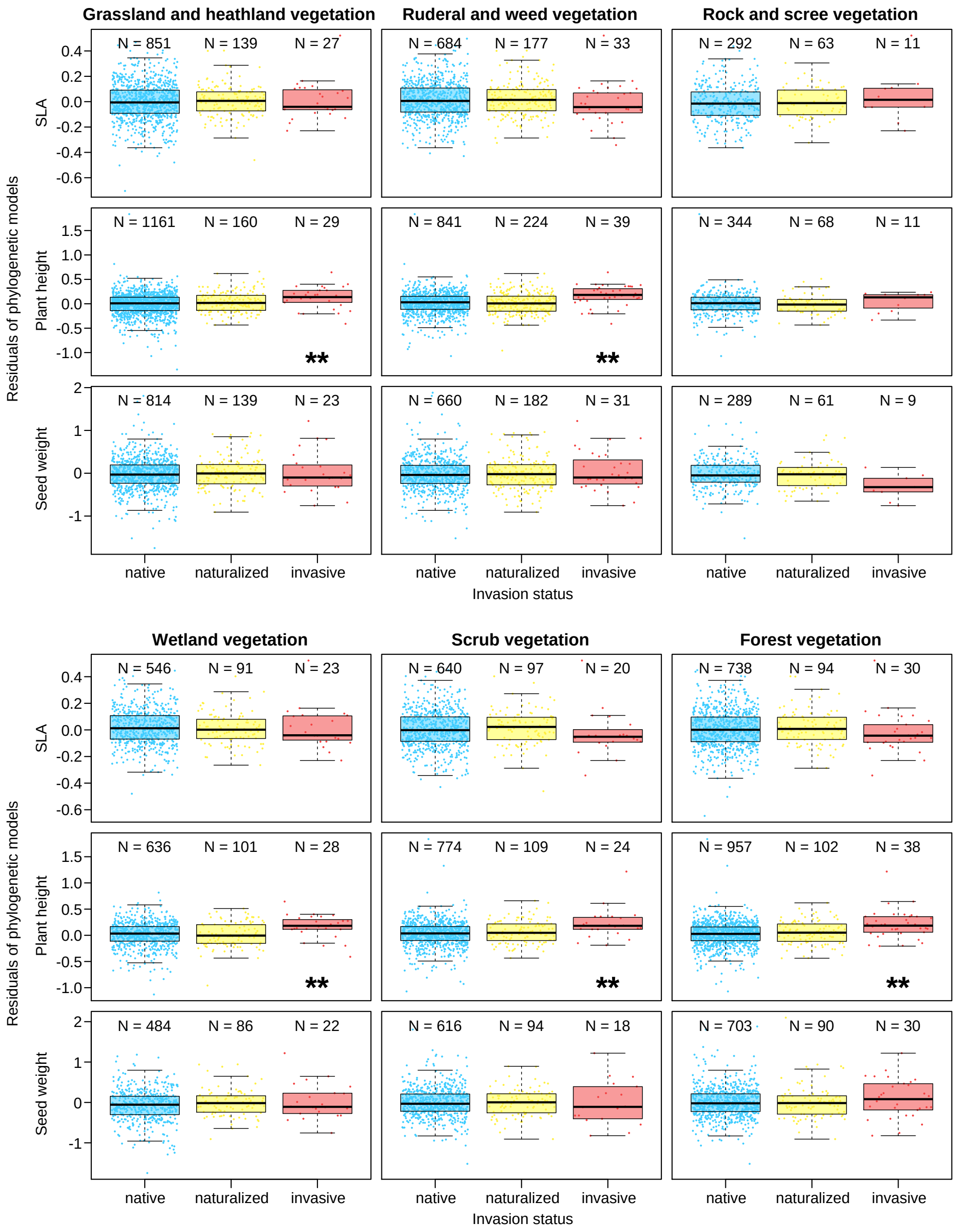

Supplementary Figure 1 | Distribution of species traits in each habitat type after accounting for phylogenetic relationships among species. The number of native, naturalized non-invasive and invasive species (N) is indicated above each boxplot. Note that species with missing trait values were removed. Asterisks below boxplots for naturalized and invasive species indicate statistical significance of their difference from native species resulting from randomization tests and adjusted using Benjamini and Hochberg's correction method[1]: *** $p \leq 0.001$; ** $0.001 < p \leq 0.01$; * $0.01 < p \leq 0.05$. For complete results of randomization tests see **Supplementary Table 1**. Thick horizontal lines in each box indicate the median. The bottom and top of each box indicate the 25th and 75th percentiles, respectively. The vertical lines (whiskers) represent either the maximum/minimum value or 1.5 × interquartile range depending on which is closer to the mean. Outliers are indicated by jittered points outside the range of whiskers.

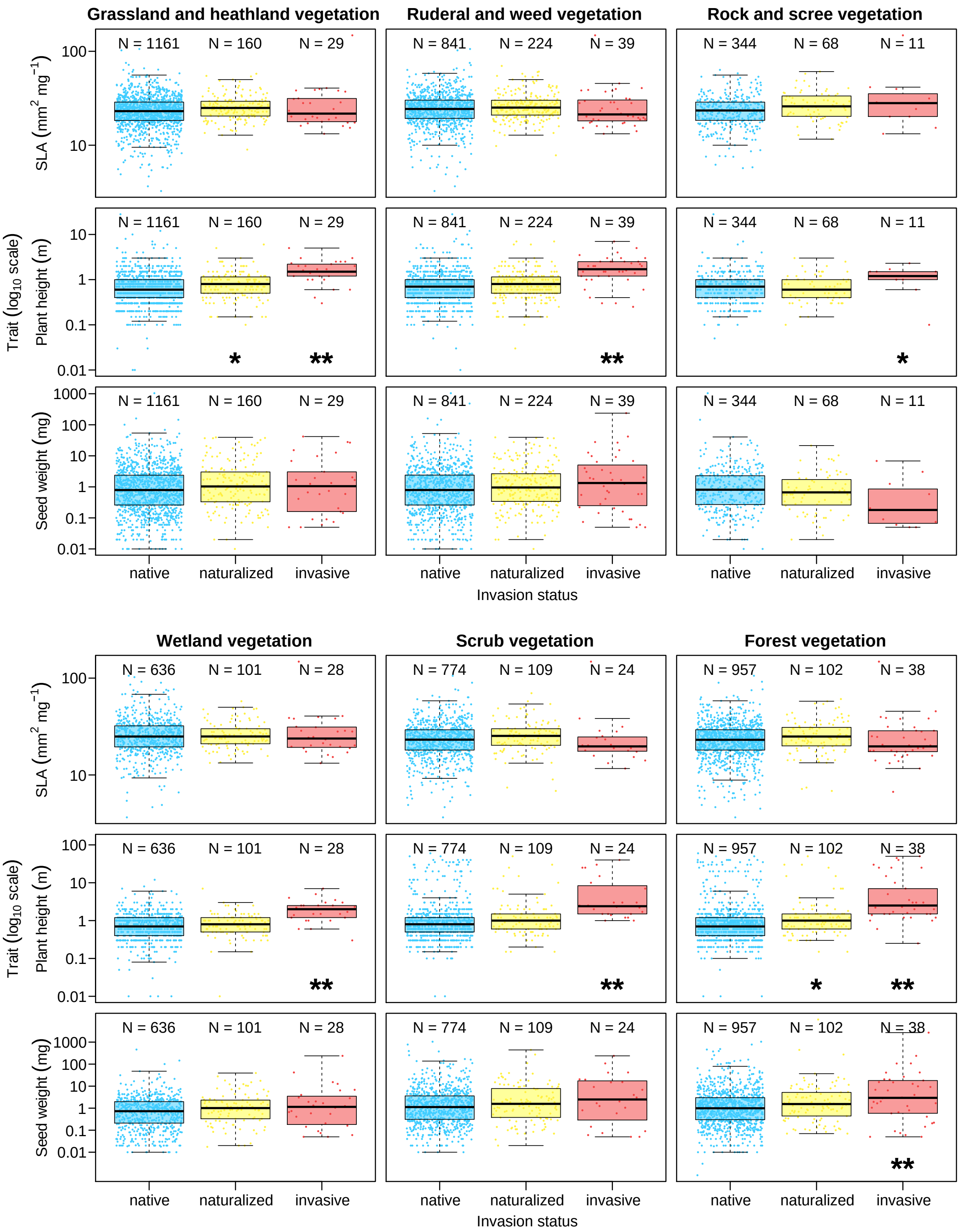

Supplementary Figure 2 | Distribution of species traits ($\log_{10}$ scale) in each habitat type after imputation of missing trait values. Missing values were imputed based on correlations among traits and species phylogenetic relatedness. For details see **Supplementary Figure 1**. Complete results of randomization tests are provided in **Supplementary Table 2**.

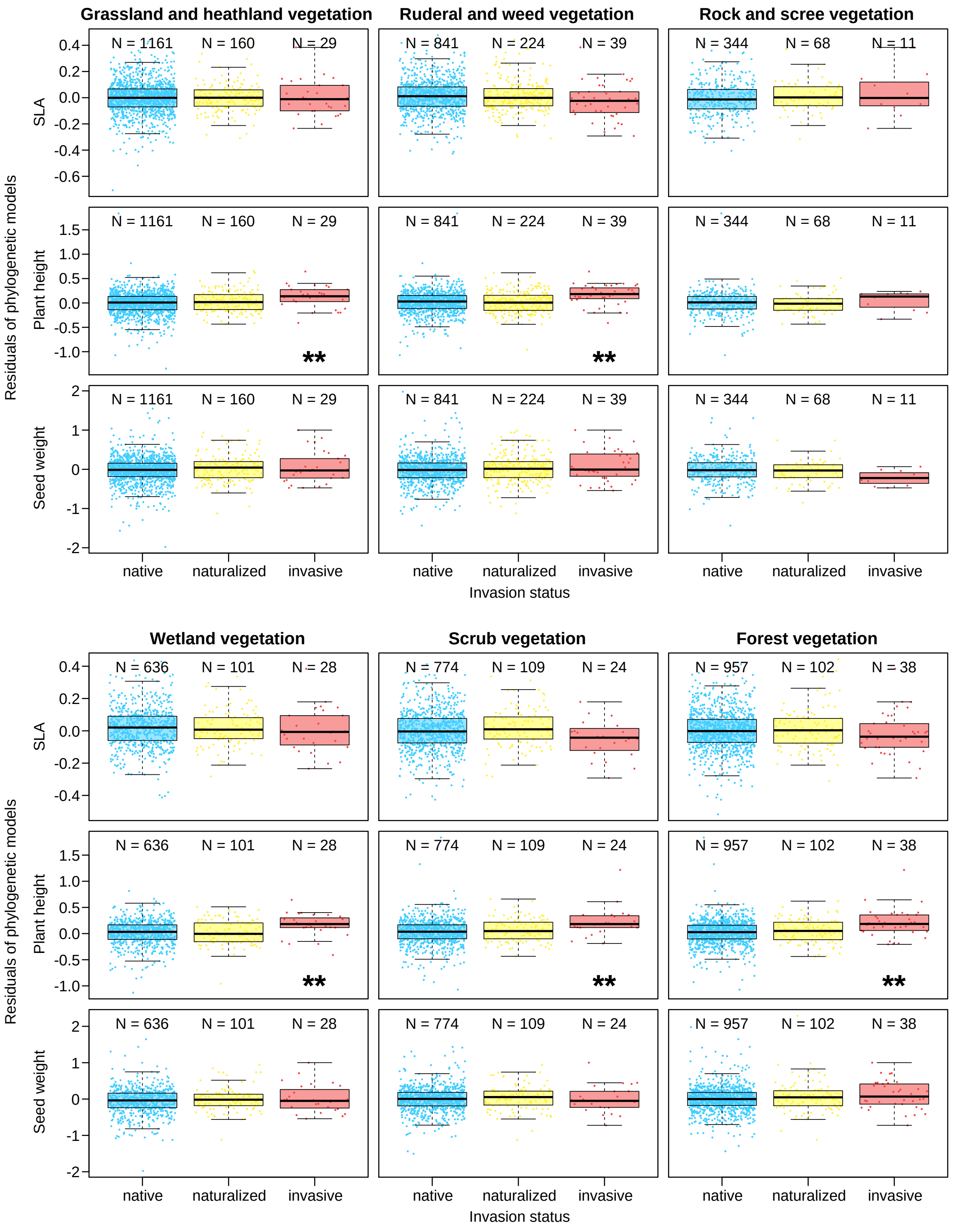

Supplementary Figure 3 | Distribution of species traits in each habitat type after imputing missing trait values and accounting for phylogenetic relationships among species. Missing values were imputed based on correlations among traits and species phylogenetic relatedness. For details see **Supplementary Figure 1**. Complete results of randomization tests are provided in **Supplementary Table 2**.

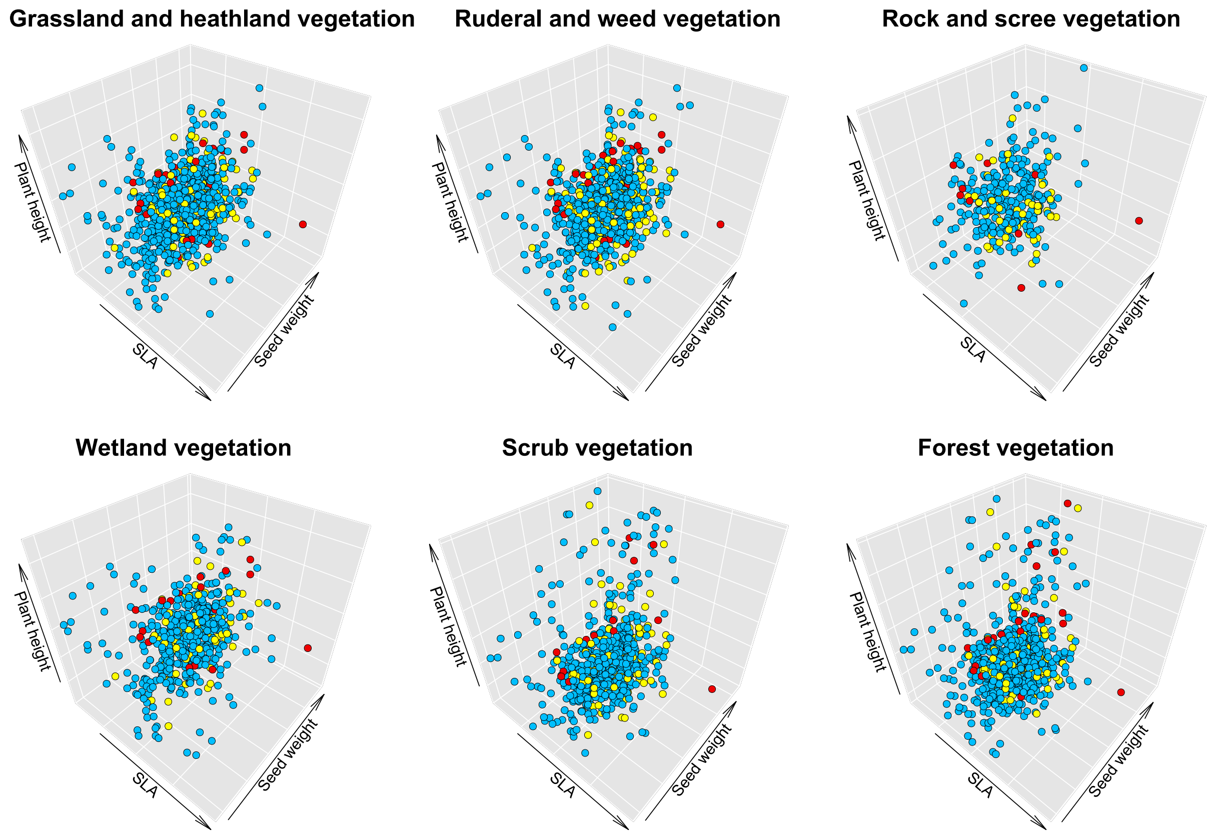

Supplementary Figure 4 | Distribution of native (blue), naturalized non-invasive (yellow) and invasive species (red) in the trait space of each habitat. Each axis represents one log_{10}-trasformed and standardized trait. Species with a missing value of any of the three traits were removed.

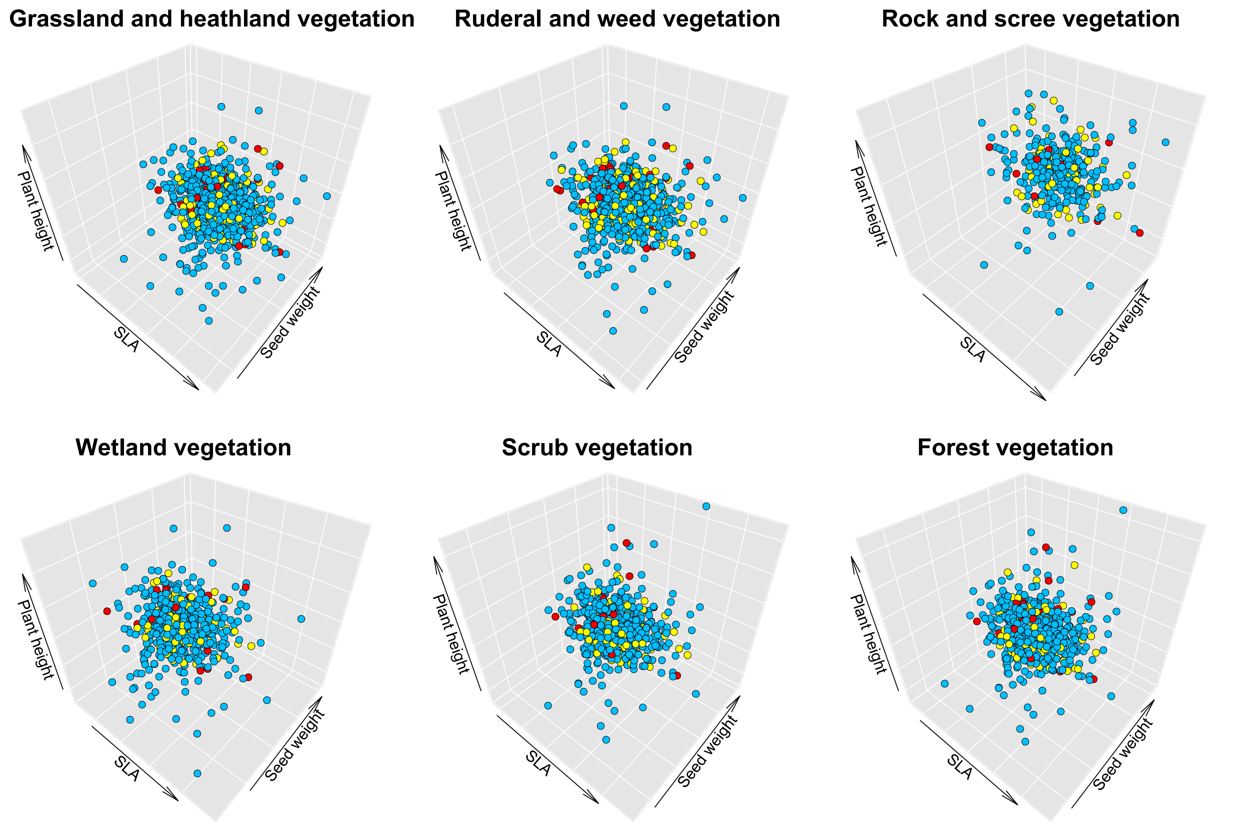

Supplementary Figure 5 | Distribution of native (blue), naturalized non-invasive (yellow) and invasive species (red) in the trait space of each habitat after accounting for phylogenetic relationships among species. Species with a missing value of any of the three traits were removed.

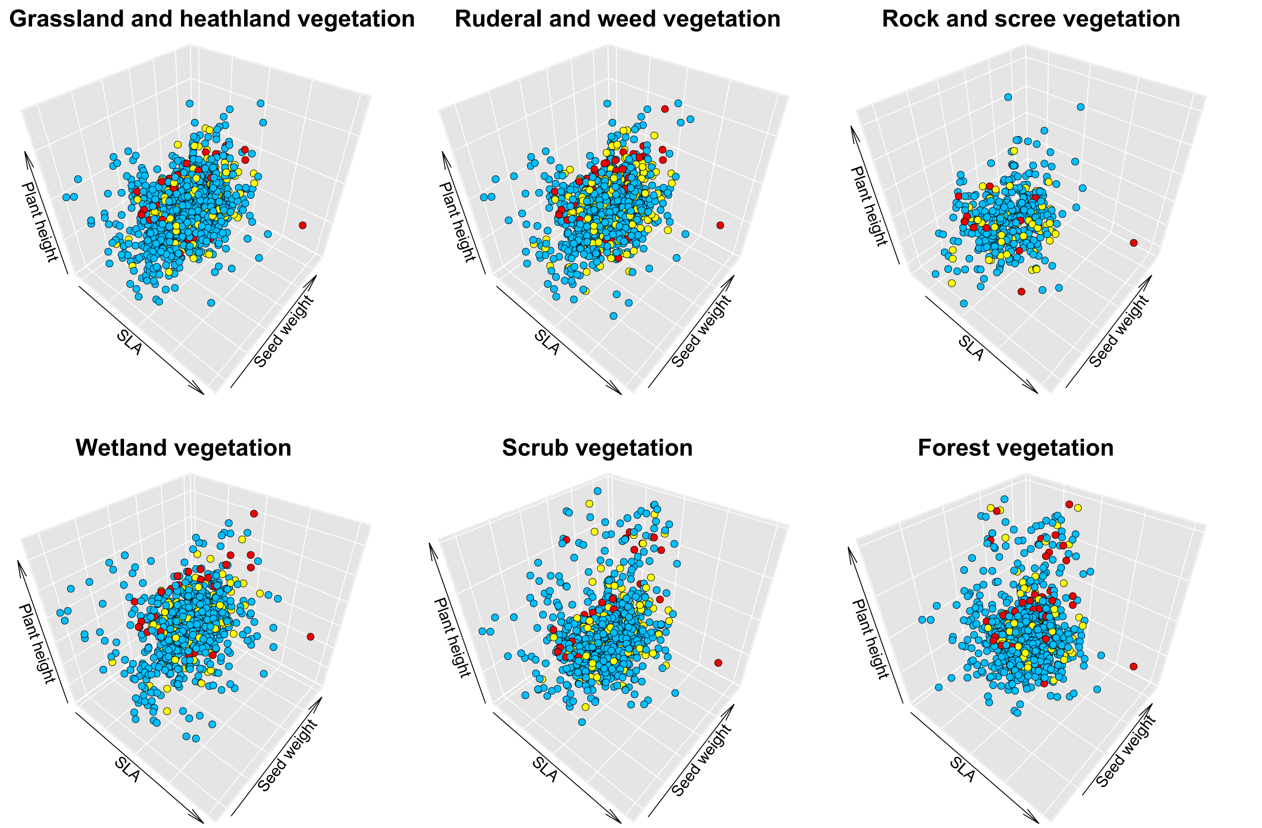

Supplementary Figure 6 | Distribution of native (blue), naturalized non-invasive (yellow) and invasive species (red) in the trait space of each habitat after imputation of missing trait values. Missing values were imputed based on correlations among traits and species phylogenetic relatedness. Each axis represents one $\log_{10}$-trasformed and standardized trait.

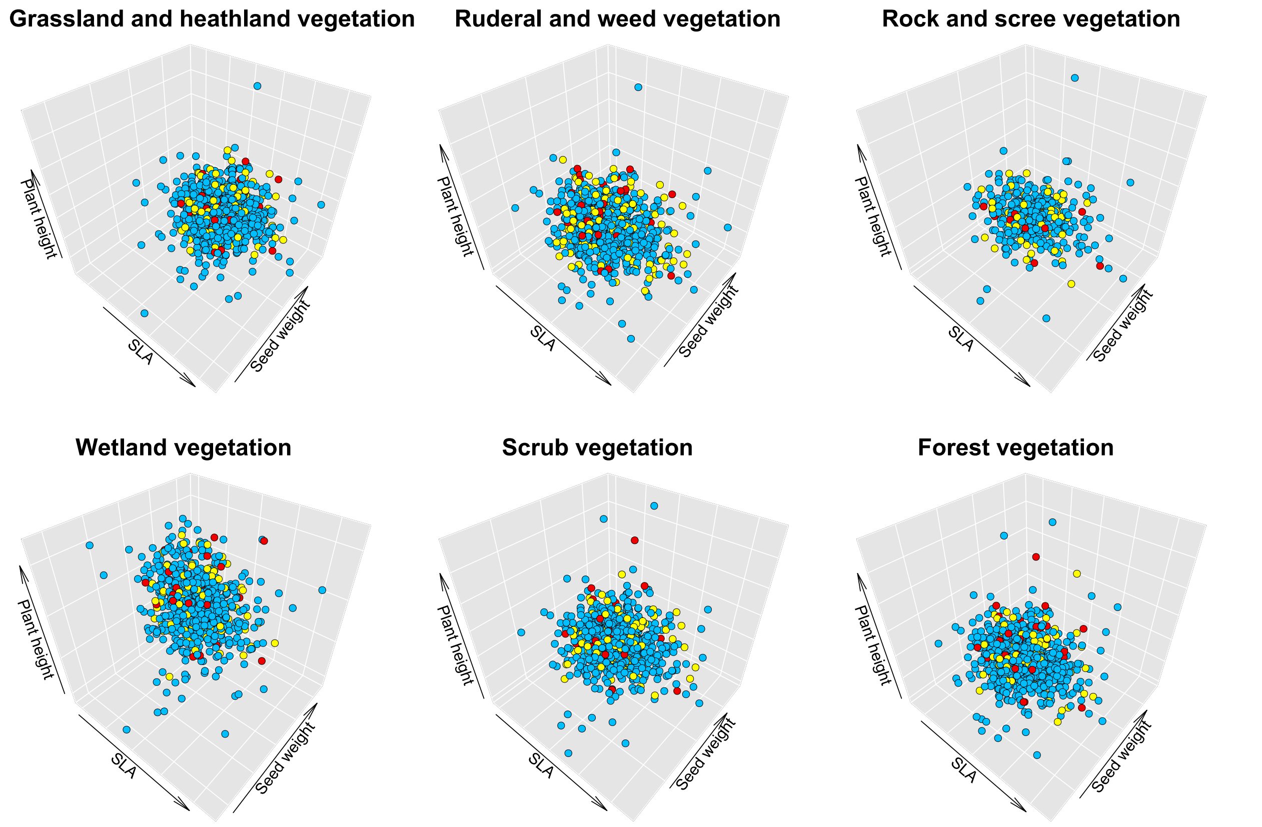

Supplementary Figure 7 | Distribution of native (blue), naturalized non-invasive (yellow) and invasive species (red) in the trait space of each habitat after imputing missing trait values and accounting for phylogenetic relationships among species. Missing trait values were imputed based on correlations among traits and species phylogenetic relatedness.

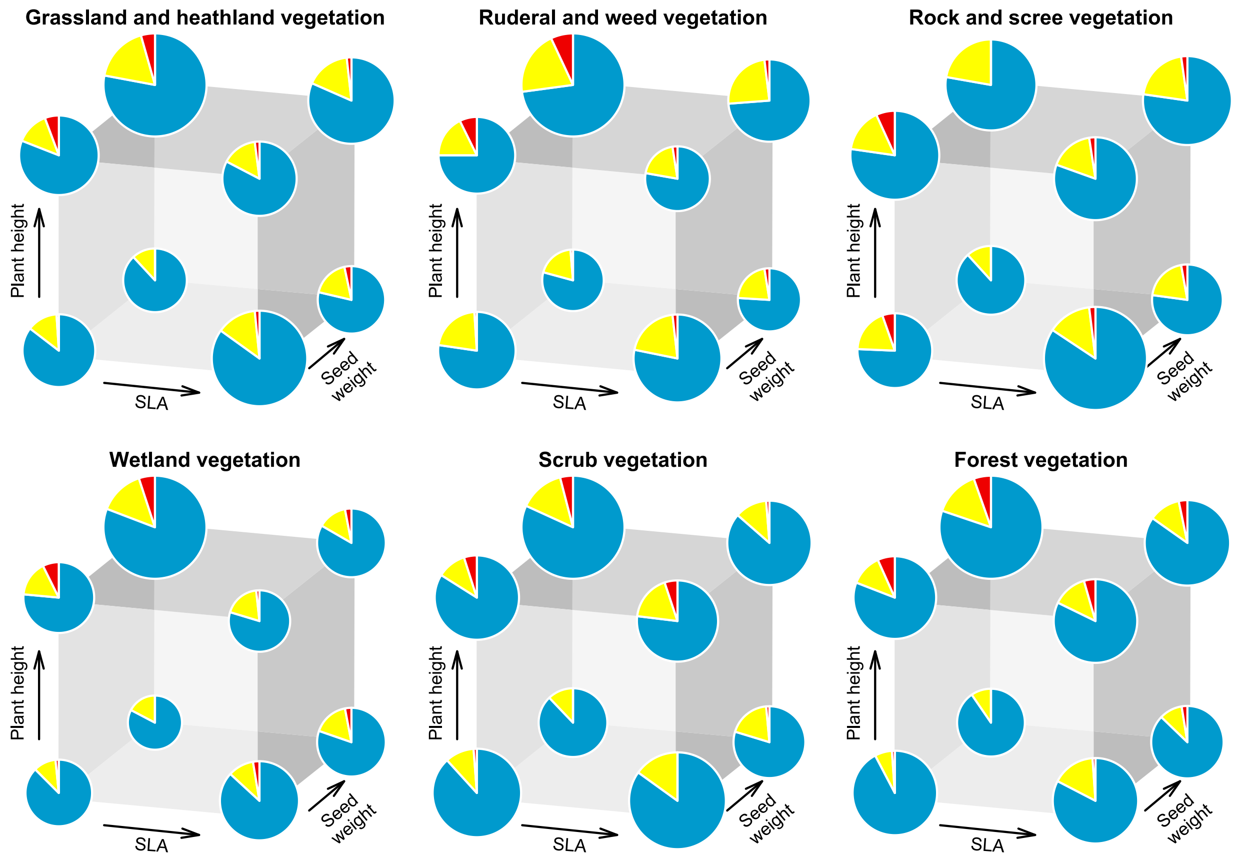

Supplementary Figure 8 | Proportion of species occupying octants of the trait space in each habitat after accounting for phylogenetic relationships among species. Blue, yellow and red colors represent, in turn, native, naturalized non-invasive, and invasive species. Octants were defined with respect to the centroid of the native species group in the trait space, i.e. by above-average or below-average SLA, plant height and seed weight of native species (**Fig. 2**). The bigger pie, the higher number of species occupies the region. For numbers of species in each octant see **Supplementary Table 4**.

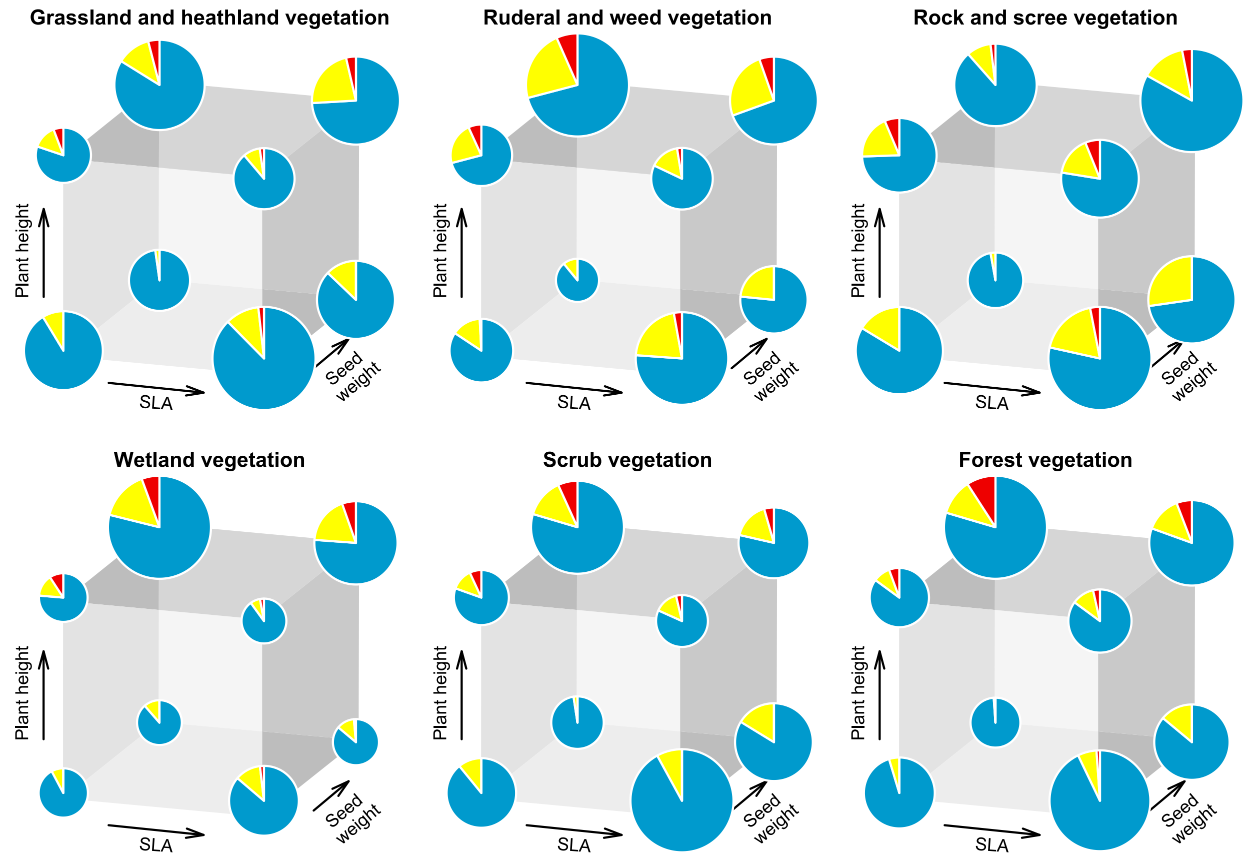

Supplementary Figure 9 | Proportion of species occupying octants of the trait space in each habitat after imputation of missing trait values. Blue, yellow and red colors represent, in turn, native, naturalized non-invasive, and invasive species. Missing trait values were imputed based on correlations among traits and species phylogenetic relatedness. Octants were defined with respect to the centroid of the native species group in the trait space, i.e. by above-average or below-average SLA, plant height and seed weight of native species (**Fig. 2**). The bigger pie, the higher number of species occupies the region. For numbers of species in each octant see **Supplementary Table 5**.

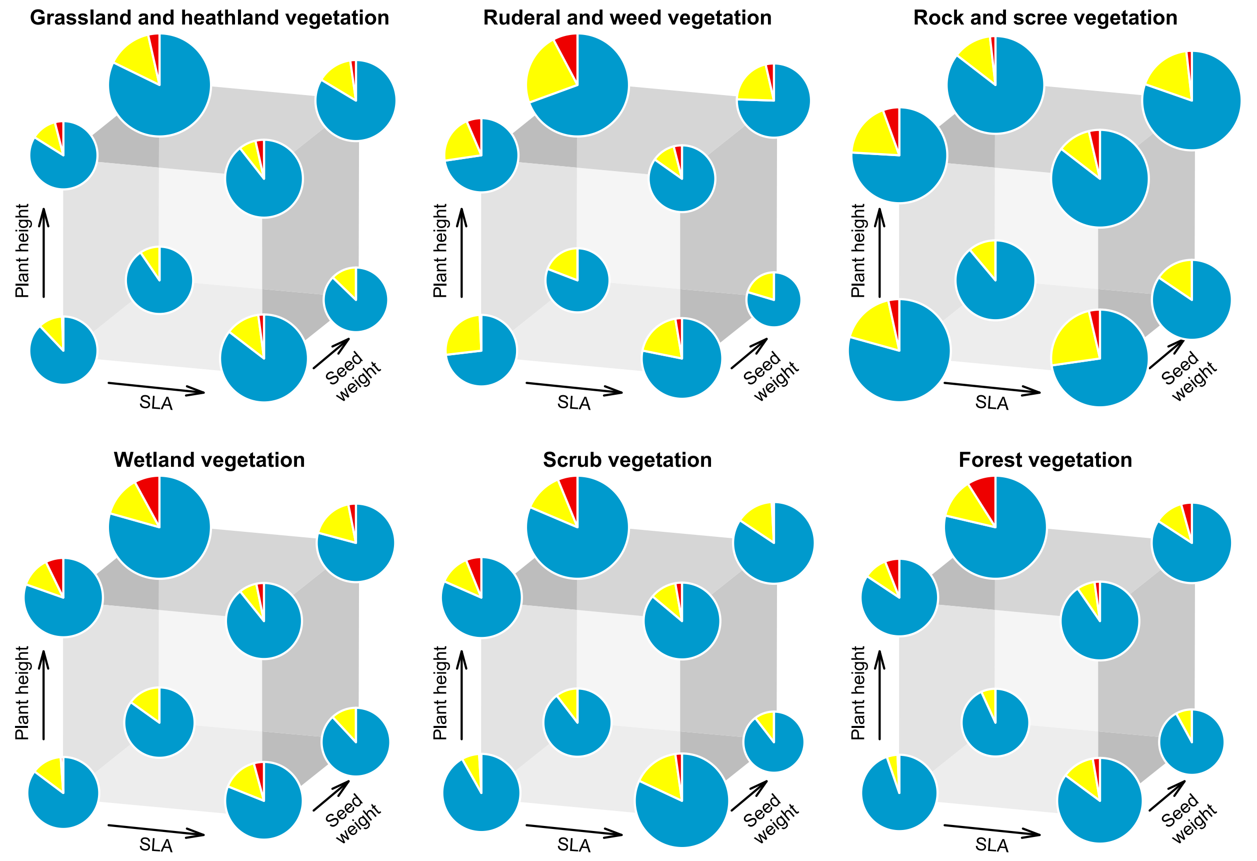

Supplementary Figure 10 | Proportion of species occupying octants of the trait space in each habitat after imputing missing trait values and accounting for phylogenetic relationships among species. Blue, yellow and red colors represent, in turn, native, naturalized non-invasive, and invasive species. Missing trait values were imputed based on correlations among traits and species phylogenetic relatedness. Octants were defined with respect to the centroid of the native species group in the trait space, i.e. by above-average or below-average SLA, plant height and seed weight of native species (**Fig. 2**). The bigger pie, the higher number of species occupies the region. For numbers of species in each octant see **Supplementary Table 6**.

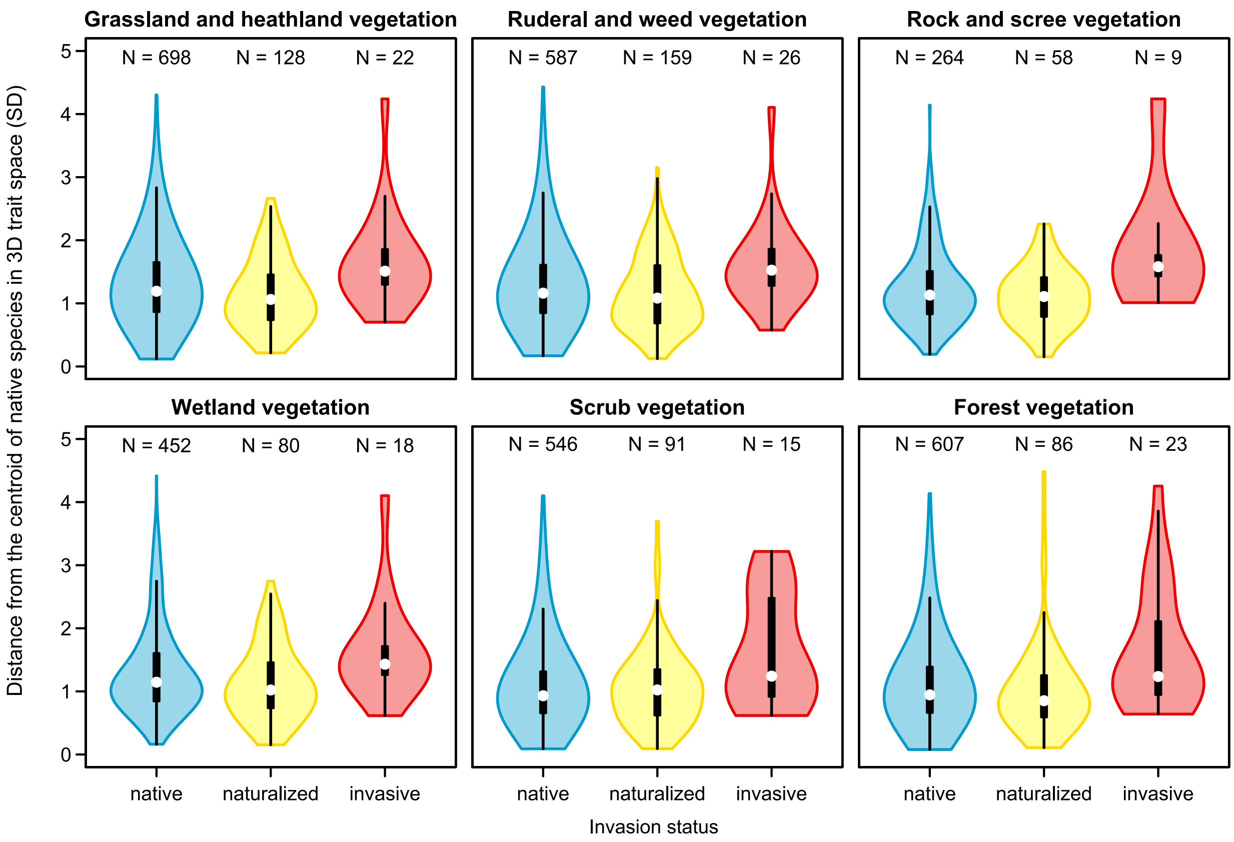

Supplementary Figure 11 | Distances of native (blue), non-invasive naturalized (yellow) and invasive (red) species from the centroid of each trait space. Species with a missing value of any of the three traits were removed from the dataset. Number of species is shown above each violin plot. White dots indicate median. The bottom and top of each black box indicate the 25th and 75th percentiles, respectively, and the vertical lines (whiskers) represent either the maximum/minimum value or 1.5 × interquartile range depending on which is closer to the mean. Outliers are not shown but they are indicated by density curves.

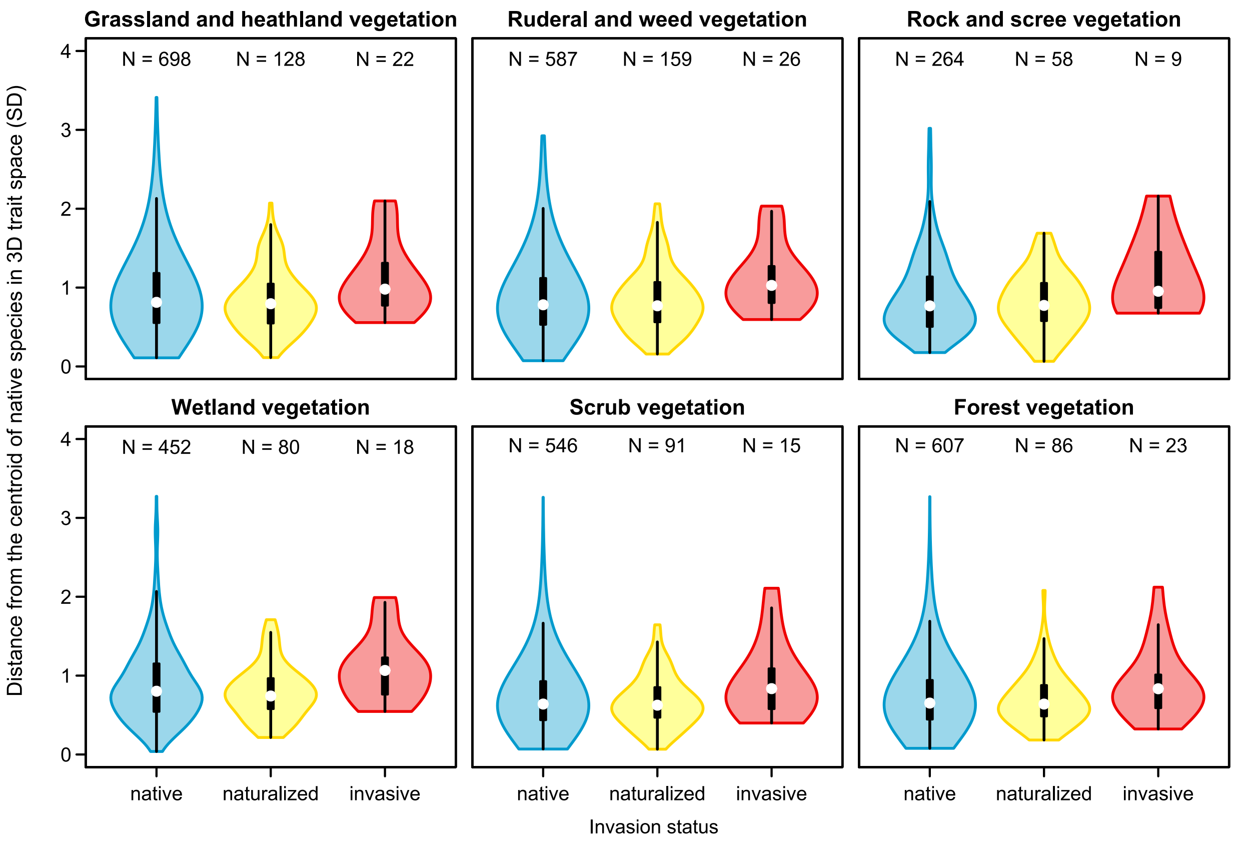

Supplementary Figure 12 | Distances of native (blue), non-invasive naturalized (yellow) and invasive (red) species from the centroid of each trait space after accounting for phylogenetic relationships among species. Species with a missing value of any of the three traits were removed from the dataset. Number of species is shown above each violin plot. For details see **Supplementary Figure 11**.

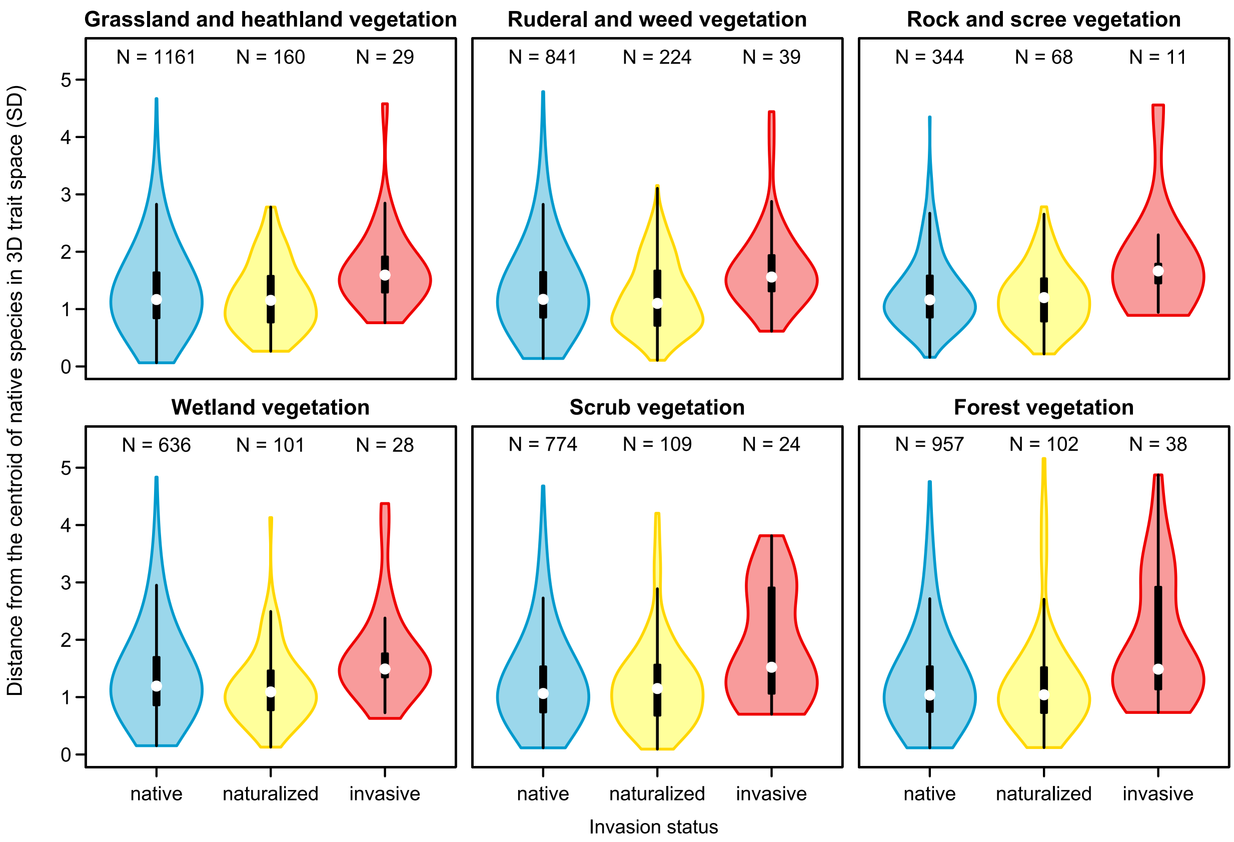

Supplementary Figure 13 | Distances of native (blue), non-invasive naturalized (yellow) and invasive (red) species from the centroid of each trait space after imputation of missing trait values. Missing values were imputed based on correlations among traits and species phylogenetic relatedness. Number of species is shown above each violin plot. For details see **Supplementary Figure 11**.

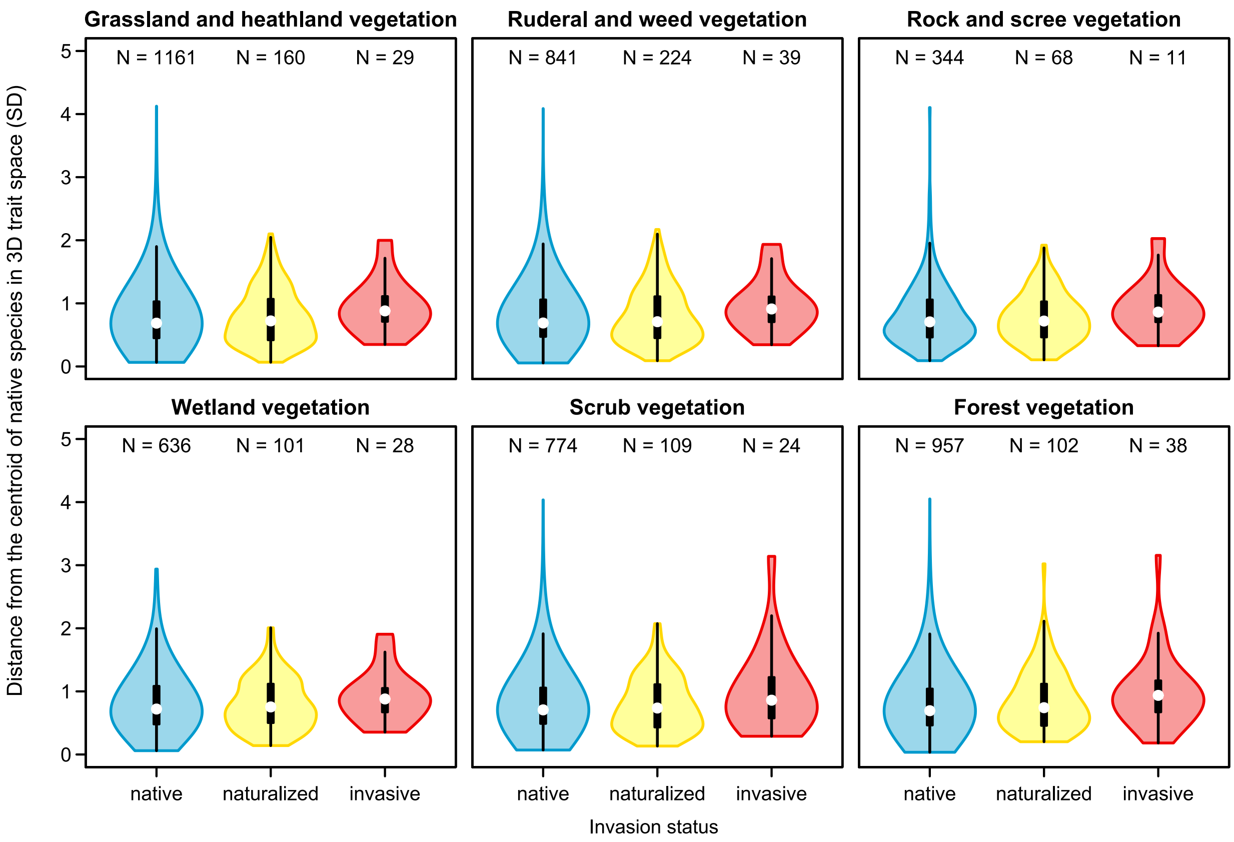

Supplementary Figure 14 | Distances of native (blue), non-invasive naturalized (yellow) and invasive (red) species from the centroid of each trait space after imputing missing trait values and accounting for phylogenetic relationships among species. Missing trait values were imputed based on correlations among traits and species phylogenetic relatedness. Number of species is shown above each violin plot. For details see **Supplementary Figure 11**.

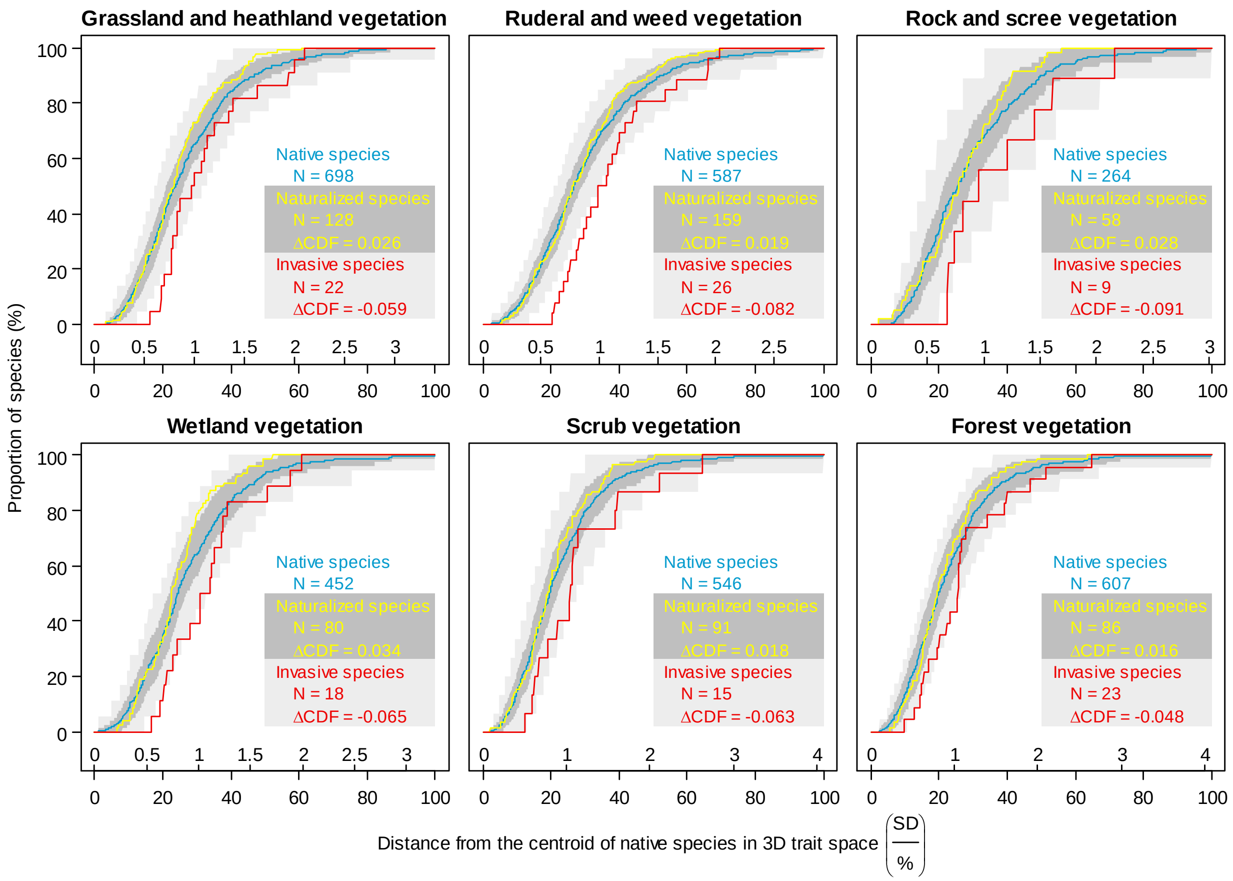

Supplementary Figure 15 | Species distribution in the trait space of each habitat after accounting for phylogenetic relationships amon species. Cumulative distribution functions (CDF) for residuals of phylogenetic models show the cumulative number of species at each distance from the centroid of the native species in the 3D trait space of each habitat. Horizontal axis shows both original distances (standard deviation units, above axis) and relative distances (%, below axis). Blue, yellow and red lines represent, in turn, native, naturalized non-invasive and invasive species. Dark grey and light grey areas show 95% confidence intervals of simulated CDFs for naturalized and invasive species, respectively. ΔCDF is the observed area between the curves calculated after scaling species distances in each habitat to relative values. Positive values indicate that the CDF for alien species (either naturalized non-invasive or invasive species) or its prevalent part is above the CDF for native species (i.e. traits of alien species are similar to the average trait of native species) whereas negative values indicate that the CDF for alien species is below the CDF for native species (i.e. traits of alien species are dissimilar to the average trait of native species). The differences between the CDFs for alien and native species were not statistically significant ($p > 0.05$). Note that species with a missing value of any of the three considered traits were removed. For complete results of randomization tests see **Supplementary Table 7**.

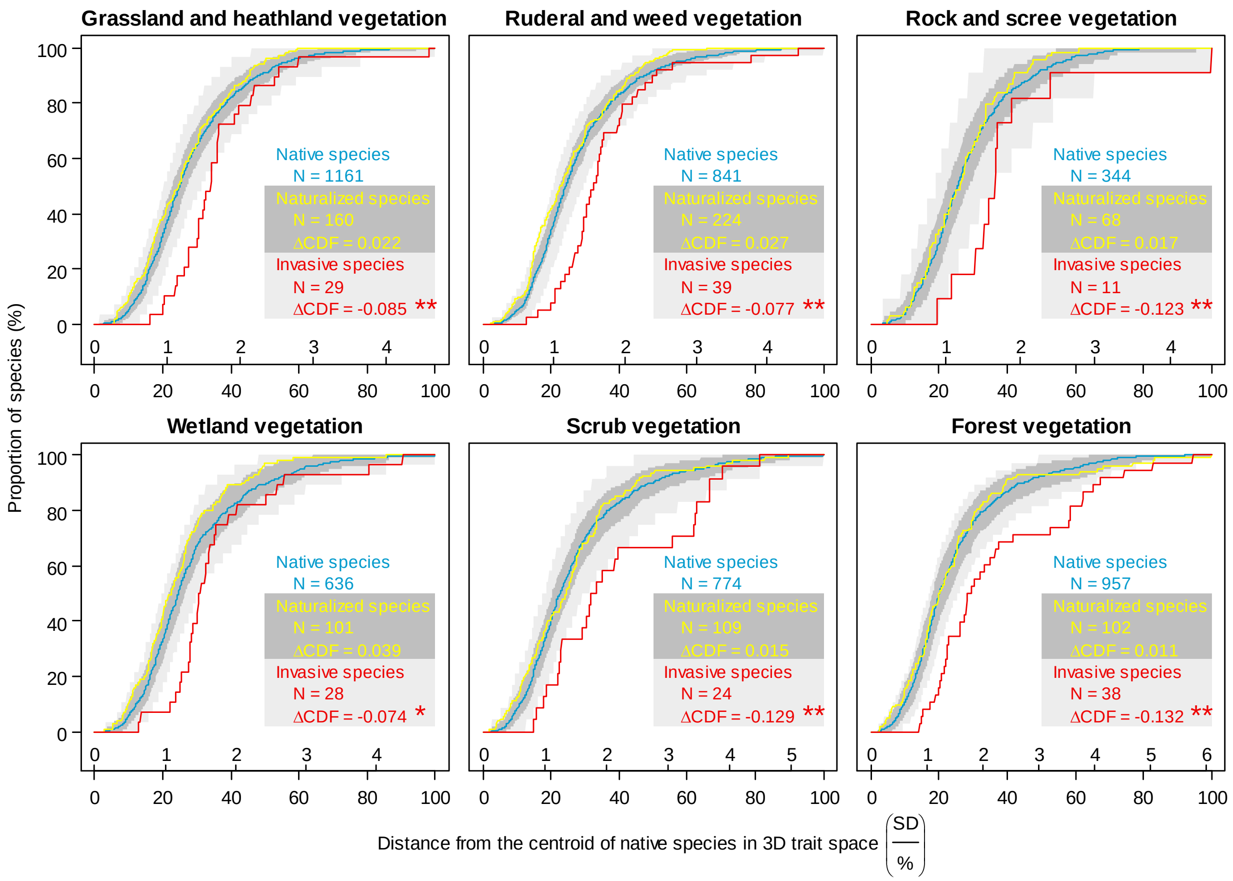

Supplementary Figure 16 | Species distribution in the trait space of each habitat after imputation of missing trait values. Cumulative distribution functions (CDF), for dataset with imputed species traits, show the cumulative number of species at each distance from the centroid of the native species in the 3D trait space of each habitat. Horizontal axis shows both original distances (standard deviation units, above axis) and relative distances (%, below axis). Statistical significance of the difference between the CDFs for alien and native species resulting from randomization test and adjusted using Benjamini and Hochberg's correction method[1] is indicated by asterisks: *** $p \leq 0.001$; ** $0.001 < p \leq 0.01$; * $0.01 < p \leq 0.05$. Missing trait values were imputed based on correlations among traits and species phylogenetic relatedness. For details see **Supplementary Figure 15**. Complete results of randomization tests are provided in **Supplementary Table 8**.

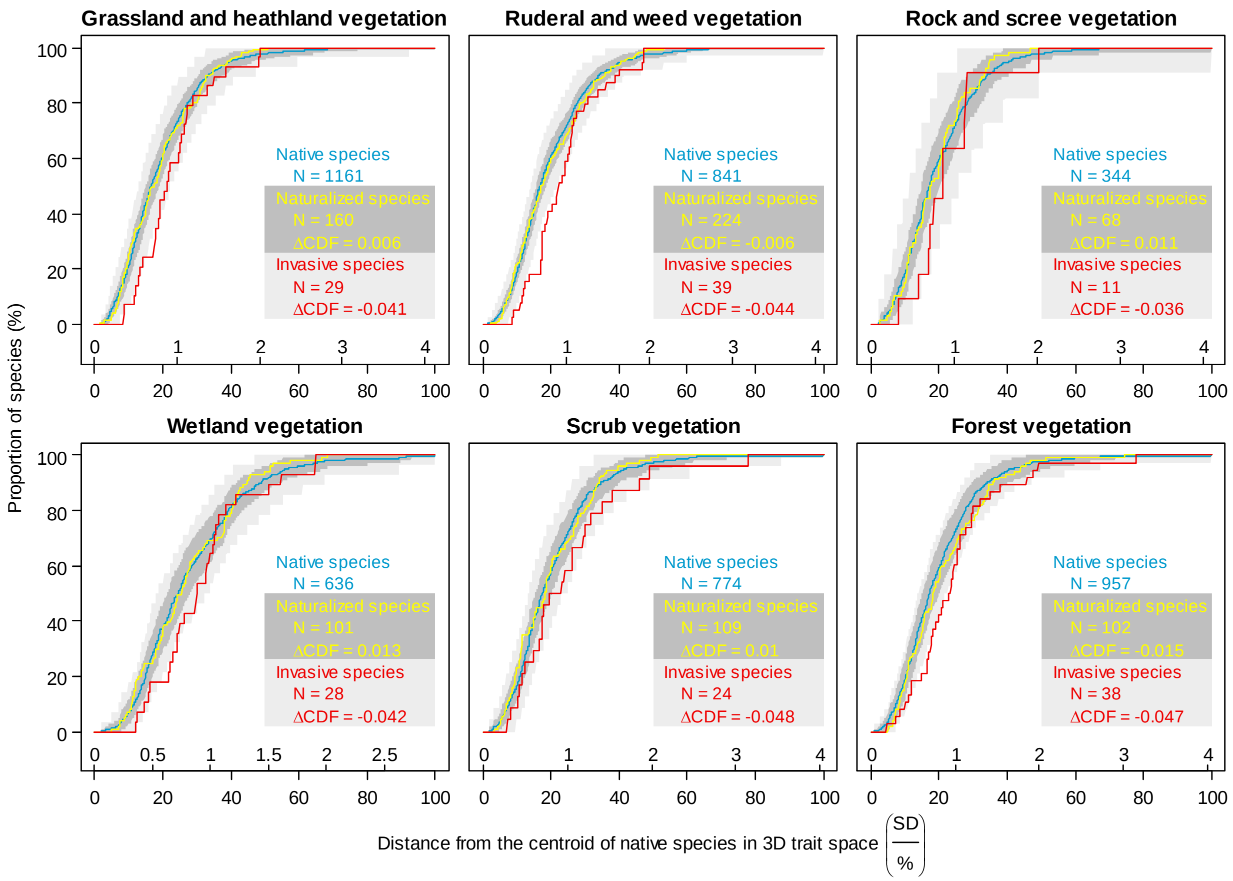

Supplementary Figure 17 | Species distribution in the trait space of each habitat after imputing missing trait values and accounting for phylogenetic relationships among species. Cumulative distribution functions (CDF), for residuals of phylogenetic models applied on dataset with imputed species traits, show the cumulative number of species at each distance from the centroid of the native species in the 3D trait space of each habitat. Horizontal axis shows both original distances (standard deviation units, above axis) and relative distances (%, below axis). The differences between the CDFs for alien and native species were not statistically significant (p > 0.05). Missing trait values were imputed based on correlations among traits and species phylogenetic relatedness. For details see **Supplementary Figure 15**. Complete results of randomization tests are provided in **Supplementary Table 8**.

Supplementary Tables

Supplementary Table 1 | Differences (Δ) of median trait values between alien (either naturalized non-invasive or invasive species) and native species across the six habitats of the Czech Republic. Species with missing trait values were removed. Results for observed traits ($mm^2\ mg^{-1}$ for SLA, m for plant height and mg for seed weight) and residuals of phylogenetic models are shown. ***p*** is a probability value resulting from randomization test and $\boldsymbol{p}_{adj.}$ is a probability value adjusted using Benjamini & Hochberg's correction method[1]. Differences that were significant after this correction are in bold.

	Specific leaf area			Plant height			Seed weight		
	Δ	*p*	$p_{adj.}$	Δ	*p*	$p_{adj.}$	Δ	*p*	$p_{adj.}$
**Observed species traits**									
Naturalized non-invasive vs. native species									
Grassland and heathland vegetation	1.90	0.043	0.106	**0.2**	**0.002**	**0.012**	0.240	0.138	0.414
Ruderal and weed vegetation	1.43	0.071	0.106	0.1	0.279	0.419	0.175	0.378	0.436
Rock and scree vegetation	2.99	0.033	0.106	-0.1	0.453	0.544	-0.170	0.436	0.436
Wetland vegetation	0.57	0.656	0.656	0.1	0.889	0.889	0.302	0.068	0.408
Scrub vegetation	2.50	0.063	0.106	0.2	0.052	0.104	0.293	0.281	0.436
Forest vegetation	1.95	0.105	0.126	**0.3**	**0.006**	**0.018**	0.260	0.370	0.436
Invasive vs. native species									
Grassland and heathland vegetation	0.28	0.932	0.932	**0.9**	**0.001**	**0.001**	0.450	0.350	0.350
Ruderal and weed vegetation	-2.68	0.206	0.286	**1.0**	**0.001**	**0.001**	0.535	0.254	0.305
Rock and scree vegetation	4.96	0.117	0.286	**0.5**	**0.021**	**0.021**	-0.630	0.132	0.198
Wetland vegetation	3.08	0.238	0.286	**1.3**	**0.001**	**0.001**	0.575	0.096	0.192
Scrub vegetation	-3.05	0.209	0.286	**1.6**	**0.001**	**0.001**	2.395	0.018	0.054
Forest vegetation	-3.27	0.125	0.286	**1.8**	**0.001**	**0.001**	**2.525**	**0.003**	**0.018**
**Residuals of phylogenetic models**									
Naturalized non-invasive vs. native species									
Grassland and heathland vegetation	0.013	0.423	0.688	0.008	0.626	0.626	0.026	0.548	0.824
Ruderal and weed vegetation	0.008	0.557	0.688	-0.023	0.206	0.486	0.018	0.595	0.824
Rock and scree vegetation	0.004	0.681	0.688	-0.027	0.324	0.486	0.025	0.687	0.824
Wetland vegetation	-0.011	0.472	0.688	-0.038	0.135	0.486	0.039	0.355	0.824
Scrub vegetation	0.024	0.159	0.688	0.012	0.526	0.626	0.035	0.517	0.824
Forest vegetation	0.006	0.688	0.688	0.024	0.259	0.486	0.006	0.942	0.942
Invasive vs. native species									
Grassland and heathland vegetation	-0.034	0.249	0.299	**0.128**	**0.006**	**0.007**	-0.069	0.324	0.425
Ruderal and weed vegetation	-0.048	0.132	0.256	**0.152**	**0.001**	**0.003**	-0.061	0.354	0.425
Rock and scree vegetation	0.030	0.521	0.521	0.120	0.094	0.094	-0.275	0.022	0.132
Wetland vegetation	-0.053	0.128	0.256	**0.148**	**0.003**	**0.006**	-0.053	0.546	0.546
Scrub vegetation	-0.050	0.171	0.256	**0.146**	**0.006**	**0.007**	-0.077	0.345	0.425
Forest vegetation	-0.044	0.155	0.256	**0.158**	**0.001**	**0.003**	0.103	0.189	0.425

Supplementary Table 2 | Differences (Δ) of median trait values between alien (either naturalized non-invasive or invasive species) and native species across the six habitats of the Czech Republic after imputation of missing trait values. Missing values were imputed based on correlations among traits and species phylogenetic relatedness. Results for observed traits ($mm^2\ mg^{-1}$ for SLA, m for plant height and mg for seed weight) and residuals of phylogenetic models are shown. $\boldsymbol{p}$ is a probability value resulting from randomization test and $\boldsymbol{p}_{adj.}$ is a probability value adjusted using Benjamini & Hochberg's correction method[1]. Differences that were significant after this correction are in bold.

	**Specific leaf area**			**Plant height**			**Seed weight**		
	Δ	p	$p_{adj.}$	Δ	p	$p_{adj.}$	Δ	p	$p_{adj.}$
**Observed species traits**									
Naturalized non-invasive vs. native species									
Grassland and heathland vegetation	1.85	0.022	0.112	**0.2**	**0.002**	**0.012**	0.242	0.166	0.249
Ruderal and weed vegetation	0.84	0.226	0.271	0.1	0.279	0.419	0.163	0.329	0.395
Rock and scree vegetation	2.50	0.058	0.112	-0.1	0.453	0.544	-0.145	0.534	0.534
Wetland vegetation	0.03	0.912	0.912	0.1	0.889	0.889	0.280	0.107	0.214
Scrub vegetation	2.16	0.057	0.112	0.2	0.052	0.104	0.446	0.105	0.214
Forest vegetation	1.86	0.075	0.112	**0.3**	**0.006**	**0.018**	0.549	0.027	0.162
Invasive vs. native species									
Grassland and heathland vegetation	-1.37	0.510	0.567	**0.9**	**0.001**	**0.001**	0.260	0.619	0.619
Ruderal and weed vegetation	-3.04	0.094	0.170	**1.0**	**0.001**	**0.001**	0.538	0.124	0.186
Rock and scree vegetation	4.64	0.113	0.170	**0.5**	**0.021**	**0.021**	-0.630	0.111	0.186
Wetland vegetation	-1.15	0.567	0.567	**1.3**	**0.001**	**0.001**	0.420	0.191	0.229
Scrub vegetation	-3.43	0.112	0.170	**1.6**	**0.001**	**0.001**	1.411	0.026	0.078
Forest vegetation	-3.36	0.039	0.170	**1.8**	**0.001**	**0.001**	**1.950**	**0.001**	**0.006**
**Residuals of phylogenetic models**									
Naturalized non-invasive vs. native species									
Grassland and heathland vegetation	0.001	0.974	0.974	0.008	0.626	0.626	0.059	0.017	0.102
Ruderal and weed vegetation	-0.012	0.225	0.518	-0.023	0.206	0.486	0.031	0.167	0.250
Rock and scree vegetation	0.016	0.339	0.518	-0.027	0.324	0.486	-0.007	0.772	0.772
Wetland vegetation	-0.013	0.302	0.518	-0.038	0.135	0.486	0.020	0.602	0.722
Scrub vegetation	0.013	0.345	0.518	0.012	0.526	0.626	0.050	0.124	0.248
Forest vegetation	0.005	0.716	0.859	0.024	0.259	0.486	0.048	0.094	0.248
Invasive vs. native species									
Grassland and heathland vegetation	-0.011	0.682	0.813	**0.128**	**0.006**	**0.007**	-0.014	0.809	0.845
Ruderal and weed vegetation	-0.035	0.111	0.248	**0.152**	**0.001**	**0.003**	0.011	0.758	0.845
Rock and scree vegetation	0.011	0.813	0.813	0.120	0.094	0.094	-0.200	0.046	0.276
Wetland vegetation	-0.027	0.328	0.492	**0.148**	**0.003**	**0.006**	-0.012	0.845	0.845
Scrub vegetation	-0.039	0.124	0.248	**0.146**	**0.006**	**0.007**	-0.054	0.415	0.830
Forest vegetation	-0.035	0.097	0.248	**0.158**	**0.001**	**0.003**	0.069	0.152	0.456

Supplementary Table 3 | Numbers of species occupying eight different regions (octants) of the trait space of each habitat. For definition of octants see **Fig. 2**.

	Octant No.							
	I	II	III	IV	V	VI	VII	VIII
**Grassland and heathland vegetation**								
Native species	108	74	61	112	72	113	81	77
Naturalized non-invasive species	34	12	14	21	20	18	8	1
Invasive species	6	1	5	6	1	3	0	0
∑	148	87	80	139	93	134	89	78
**Ruderal and weed vegetation**								
Native species	91	68	58	106	61	87	70	46
Naturalized non-invasive species	34	14	18	28	24	27	10	4
Invasive species	6	1	5	9	1	3	1	0
∑	131	83	81	143	86	117	81	50
**Rock and scree vegetation**								
Native species	45	34	30	39	26	33	34	23
Naturalized non-invasive species	8	8	8	5	12	12	5	0
Invasive species	2	1	3	1	0	2	0	0
∑	55	43	41	45	38	47	39	23
**Wetland vegetation**								
Native species	67	39	41	84	55	77	45	44
Naturalized non-invasive species	19	5	7	15	13	12	7	2
Invasive species	6	1	4	4	1	2	0	0
∑	92	45	52	103	69	91	52	46
**Scrub vegetation**								
Native species	68	40	42	80	71	110	80	55
Naturalized non-invasive species	16	10	7	14	20	15	8	1
Invasive species	5	1	3	6	0	0	0	0
∑	89	51	52	100	91	125	88	56
**Forest vegetation**								
Native species	77	54	55	89	79	111	80	62
Naturalized non-invasive species	15	12	7	14	20	10	7	1
Invasive species	7	1	4	10	0	1	0	0
∑	99	67	66	113	99	122	87	63

Supplementary Table 4 | Numbers of species occupying eight different regions (octants) of the trait space of each habitat after accounting for phylogenetic relationships among species. For definition of octants see **Fig. 2**.

	Octant No.							
	I	II	III	IV	V	VI	VII	VIII
**Grassland and heathland vegetation**								
Native species	93	81	85	106	70	107	82	74
Naturalized non-invasive species	19	15	14	24	16	17	13	10
Invasive species	2	2	6	6	3	2	1	0
∑	114	98	105	136	89	126	96	84
**Ruderal and weed vegetation**								
Native species	76	63	72	94	60	86	75	61
Naturalized non-invasive species	25	16	17	26	17	22	21	15
Invasive species	2	2	7	9	2	2	1	1
∑	103	81	96	129	79	110	97	77
**Rock and scree vegetation**								
Native species	34	33	34	35	27	43	28	30
Naturalized non-invasive species	9	7	7	10	7	7	7	4
Invasive species	1	1	3	0	1	1	2	0
∑	44	41	44	45	35	51	37	34
**Wetland vegetation**								
Native species	55	47	52	80	53	66	56	43
Naturalized non-invasive species	9	11	11	14	11	8	7	9
Invasive species	2	1	5	5	2	2	1	0
∑	66	59	68	99	66	76	64	52
**Scrub vegetation**								
Native species	70	60	68	81	55	79	75	58
Naturalized non-invasive species	10	14	9	14	13	14	9	8
Invasive species	1	4	4	4	1	0	1	0
∑	81	78	81	99	69	93	85	66
**Forest vegetation**								
Native species	78	74	72	89	68	76	84	66
Naturalized non-invasive species	11	12	11	16	8	15	6	7
Invasive species	3	4	6	6	2	1	1	0
∑	92	90	89	111	78	92	91	73

Supplementary Table 5 | Numbers of species occupying eight different regions (octants) of the trait space of each habitat. Missing trait values were imputed based on correlations among traits and species phylogenetic relatedness. For definition of octants see **Fig. 2**.

	Octant No.							
	I	II	III	IV	V	VI	VII	VIII
**Grassland and heathland vegetation**								
Native species	143	119	97	166	149	198	158	131
Naturalized non-invasive species	43	12	17	24	22	24	15	3
Invasive species	7	3	7	8	0	4	0	0
∑	193	134	121	198	171	226	173	134
**Ruderal and weed vegetation**								
Native species	116	96	83	139	98	134	102	73
Naturalized non-invasive species	42	18	26	44	30	37	18	9
Invasive species	9	3	8	13	0	5	1	0
∑	167	117	117	196	128	176	121	82
**Rock and scree vegetation**								
Native species	54	38	35	46	40	51	46	34
Naturalized non-invasive species	9	8	9	5	15	12	9	1
Invasive species	2	3	3	1	0	2	0	0
∑	65	49	47	52	55	65	55	35
**Wetland vegetation**								
Native species	99	63	58	127	62	94	71	62
Naturalized non-invasive species	24	5	11	25	9	13	6	8
Invasive species	7	2	7	9	1	2	0	0
∑	130	70	76	161	72	109	77	70
**Scrub vegetation**								
Native species	88	67	70	117	103	150	98	81
Naturalized non-invasive species	19	12	11	20	20	13	12	2
Invasive species	5	3	6	10	0	0	0	0
∑	112	82	87	147	123	163	110	83
**Forest vegetation**								
Native species	124	97	91	148	117	169	122	89
Naturalized non-invasive species	21	13	10	21	19	11	6	1
Invasive species	9	4	6	17	0	2	0	0
∑	154	114	107	186	136	182	128	90

Supplementary Table 6 | Numbers of species occupying eight different regions (octants) of the trait space of each habitat after imputing missing trait values and accounting for phylogenetic relationships among species. Missing trait values were imputed based on correlations among traits and species phylogenetic relatedness. For definition of octants see **Fig. 2**.

	Octant No.							
	I	II	III	IV	V	VI	VII	VIII
**Grassland and heathland vegetation**								
Native species	148	152	125	185	123	164	131	133
Naturalized non-invasive species	25	12	18	32	18	24	17	14
Invasive species	4	6	6	8	0	4	1	0
∑	177	170	149	225	141	192	149	147
**Ruderal and weed vegetation**								
Native species	105	106	101	134	82	118	98	97
Naturalized non-invasive species	29	14	29	44	21	29	35	23
Invasive species	5	5	9	15	0	4	1	0
∑	139	125	139	193	103	151	134	120
**Rock and scree vegetation**								
Native species	45	47	41	47	38	40	46	40
Naturalized non-invasive species	10	6	10	7	7	13	10	5
Invasive species	1	2	3	1	0	2	2	0
∑	56	55	54	55	45	55	58	45
**Wetland vegetation**								
Native species	76	83	78	100	74	77	75	73
Naturalized non-invasive species	17	7	12	16	10	14	12	13
Invasive species	3	3	7	10	0	4	1	0
∑	96	93	97	126	84	95	88	86
**Scrub vegetation**								
Native species	97	93	93	118	77	109	101	86
Naturalized non-invasive species	17	12	14	18	9	21	8	10
Invasive species	1	3	7	9	0	3	1	0
∑	115	108	114	145	86	133	110	96
**Forest vegetation**								
Native species	117	123	113	140	104	126	125	109
Naturalized non-invasive species	16	10	13	22	9	18	6	8
Invasive species	6	3	8	16	0	4	1	0
∑	139	136	134	178	113	148	132	117

Supplementary Table 7 | Differences between the cumulative distribution functions (CDFs) of alien (either naturalized non-invasive or invasive species) and native species. **∆CDF** is the observed area between the curves. Positive values indicate that the CDF for alien species or its prevalent part is above the CDF for native species whereas negative values indicate that the CDF for alien species is below the CDF for native species. ***p*** is a probability value resulting from randomization test and $\boldsymbol{p_{adj.}}$ is a probability value adjusted using Benjamini and Hochberg's correction method[1]. Differences that were significant after this correction are in bold. Note that species with missing trait values were removed.

	Naturalized species			Invasive species		
	∆CDF	*p*	$p_{adj.}$	∆CDF	*p*	$p_{adj.}$
**Observed species traits**						
Grassland and heathland vegetation	**0.041**	**0.006**	**0.036**	**-0.077**	**0.020**	**0.030**
Ruderal and weed vegetation	0.030	0.043	0.092	**-0.069**	**0.027**	**0.032**
Rock and scree vegetation	0.032	0.147	0.176	**-0.146**	**0.005**	**0.014**
Wetland vegetation	0.040	0.046	0.092	-0.075	0.064	0.064
Scrub vegetation	0.019	0.346	0.346	**-0.127**	**0.007**	**0.014**
Forest vegetation	0.027	0.142	0.176	**-0.112**	**0.003**	**0.014**
**Residuals of phylogenetic models**						
Grassland and heathland vegetation	0.026	0.082	0.246	-0.059	0.075	0.124
Ruderal and weed vegetation	0.019	0.249	0.344	-0.082	0.021	0.124
Rock and scree vegetation	0.028	0.273	0.344	-0.091	0.153	0.153
Wetland vegetation	0.034	0.077	0.246	-0.065	0.073	0.124
Scrub vegetation	0.018	0.287	0.344	-0.063	0.083	0.124
Forest vegetation	0.016	0.362	0.362	-0.048	0.106	0.127

Supplementary Table 8 | Differences between the CDFs of alien (either naturalized non-invasive or invasive species) and native species. Missing trait values were imputed based on correlations among traits and species phylogenetic relatedness. **ΔCDF** shows observed area between the curves. Positive values indicate that the CDF for non-native species or its prevalent part is above the CDF for native species whereas negative values indicate that the CDF for non-native species is below the CDF for native species. ***p*** is a probability value resulting from randomization test and $\boldsymbol{p_{adj.}}$ is a probability value adjusted using Benjamini and Hochberg's correction method[1]. Differences that were significant after this correction are in bold.

	Naturalized species			Invasive species		
	**ΔCDF**	***P***	$\boldsymbol{P_{adj.}}$	**ΔCDF**	***P***	$\boldsymbol{P_{adj.}}$
**Observed species traits**						
Grassland and heathland vegetation	0.022	0.091	0.182	**-0.085**	**0.002**	**0.003**
Ruderal and weed vegetation	0.027	0.024	0.072	**-0.077**	**0.001**	**0.003**
Rock and scree vegetation	0.017	0.520	0.624	**-0.123**	**0.008**	**0.010**
Wetland vegetation	0.039	0.024	0.072	**-0.074**	**0.021**	**0.021**
Scrub vegetation	0.015	0.506	0.624	**-0.129**	**0.002**	**0.003**
Forest vegetation	0.011	0.687	0.687	**-0.132**	**0.001**	**0.003**
**Residuals of phylogenetic models**						
Grassland and heathland vegetation	0.006	0.711	0.711	-0.041	0.086	0.129
Ruderal and weed vegetation	-0.006	0.592	0.711	-0.044	0.019	0.072
Rock and scree vegetation	0.011	0.656	0.711	-0.036	0.427	0.427
Wetland vegetation	0.013	0.622	0.711	-0.042	0.194	0.233
Scrub vegetation	0.010	0.501	0.711	-0.048	0.080	0.129
Forest vegetation	-0.015	0.250	0.711	-0.047	0.024	0.072

Supplementary Table 9 | Numbers of species with available trait values. Percentage proportion is given in parentheses. See **Table 1** for total numbers of species in each habitat type.

Number of species with available trait values (percentage proportion)			
	**native**	**naturalized**	**invasive**
**Specific leaf area**			
Grassland and heathland vegetation	851 (73)	139 (87)	27 (93)
Ruderal and weed vegetation	684 (81)	177 (79)	33 (85)
Rock and scree vegetation	292 (85)	63 (93)	11 (100)
Wetland vegetation	546 (86)	91 (90)	23 (82)
Scrub vegetation	640 (83)	97 (89)	20 (83)
Forest vegetation	738 (77)	94 (92)	30 (79)
**Plant height**			
Grassland and heathland vegetation	1161 (100)	160 (100)	29 (100)
Ruderal and weed vegetation	841 (100)	224 (100)	39 (100)
Rock and scree vegetation	344 (100)	68 (100)	11 (100)
Wetland vegetation	636 (100)	101 (100)	28 (100)
Scrub vegetation	774 (100)	109 (100)	24 (100)
Forest vegetation	957 (100)	102 (100)	38 (100)
**Seed weight**			
Grassland and heathland vegetation	814 (70)	139 (87)	23 (79)
Ruderal and weed vegetation	660 (78)	182 (81)	31 (79)
Rock and scree vegetation	289 (84)	61 (90)	9 (82)
Wetland vegetation	484 (76)	86 (85)	22 (79)
Scrub vegetation	616 (80)	94 (86)	18 (75)
Forest vegetation	703 (73)	90 (88)	30 (79)
**All three traits**			
Grassland and heathland vegetation	698 (60)	128 (80)	22 (76)
Ruderal and weed vegetation	587 (70)	159 (71)	26 (67)
Rock and scree vegetation	264 (77)	58 (85)	9 (82)
Wetland vegetation	452 (71)	80 (79)	18 (64)
Scrub vegetation	546 (71)	91 (83)	15 (63)
Forest vegetation	607 (63)	86 (84)	23 (61)

Supplementary References

1. Benjamini, Y. & Hochberg, Y. Controlling the false discovery rate: a practical and powerful approach to multiple testing. *J. R. Stat. Soc. Ser. B Methodol.* **57,** 289–300 (1995).
