# Supplementary material for: Similarity of introduced plant species to native ones facilitates naturalization, but differences enhance invasion success
Source: Nat Commun. 2018 Nov 6;9:4631. doi: 10.1038/s41467-018-06995-4 (PMC6219509; doi:10.1038/s41467-018-06995-4)
Supplement: Supplementary file 3 — Description of Additional Supplementary Files [file 41467_2018_6995_MOESM3_ESM.pdf]

Description of Additional Supplementary Files

File Name: Supplementary Data
Description: Contains list of all species used in the analyses
