# Supplementary material for: Similarity of introduced plant species to native ones facilitates naturalization, but differences enhance invasion success
Source: Nat Commun. 2018 Nov 6;9:4631. doi: 10.1038/s41467-018-06995-4 (PMC6219509; doi:10.1038/s41467-018-06995-4)
Supplement: Supplementary file 5 — Reporting Summary [file 41467_2018_6995_MOESM5_ESM.docx]

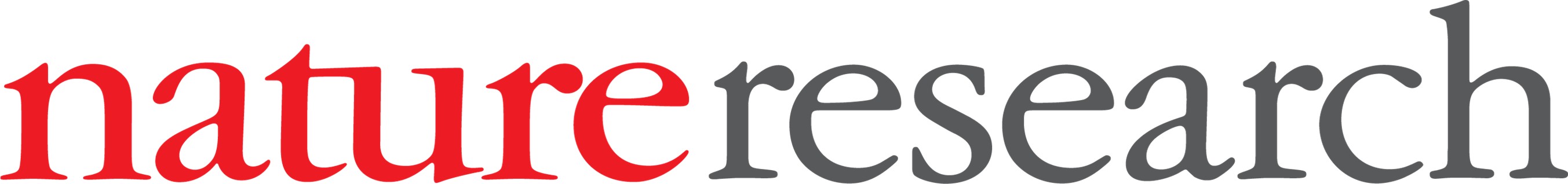
Corresponding author(s):

Jane Molofsky, Department of Plant Biology, University of Vermont, Burlington, VT 05405, [USA. Jane.Molofsky@uvm.edu](mailto:Jane.Molofsky@uvm.edu)

1

nature research | reporting summary

Initial submission Revised version Final submission

Reporting Summary

Nature Research wishes to improve the reproducibility of the work that we publish. This form provides structure for consistency and transparency in reporting. For further information on Nature Research policies, see [Authors & Referees](http://www.nature.com/authors/index.html) and the [Editorial Policy Checklist](https://www.nature.com/authors/policies/Policy.pdf).

Please do not complete any field with "not applicable" or n/a. Refer to the help text for what text to use if an item is not relevant to your study. For final submission: please carefully check your responses for accuracy; you will not be able to make changes later.

## Statistical parameters

When statistical analyses are reported, confirm that the following items are present in the relevant location (e.g. figure legend, table legend, main text, or Methods section).

n/a Confirmed


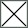

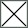


The exact sample size (*n*) for each experimental group/condition, given as a discrete number and unit of measurement

An indication of whether measurements were taken from distinct samples or whether the same sample was measured repeatedly The statistical test(s) used AND whether they are one- or two-sided

*Only common tests should be described solely by name; describe more complex techniques in the Methods section.*

A description of all covariates tested

A description of any assumptions or corrections, such as tests of normality and adjustment for multiple comparisons

A full description of the statistics including central tendency (e.g. means) or other basic estimates (e.g. regression coefficient) AND variation (e.g. standard deviation) or associated estimates of uncertainty (e.g. confidence intervals)

For null hypothesis testing, the test statistic (e.g. *F*, *t*, *r*) with confidence intervals, effect sizes, degrees of freedom and *P* value noted

*Give P values as exact values whenever suitable.*

For Bayesian analysis, information on the choice of priors and Markov chain Monte Carlo settings

For hierarchical and complex designs, identification of the appropriate level for tests and full reporting of outcomes Estimates of effect sizes (e.g. Cohen's *d*, Pearson's *r*), indicating how they were calculated

Clearly defined error bars

*State explicitly what error bars represent (e.g. SD, SE, CI)*

*Our web collection on* [*statistics for biologists*](http://www.nature.com/collections/qghhqm) *may be useful.*

## Software and code

Policy information about [availability of computer code](http://www.nature.com/authors/policies/availability.html#code) Data collection

Phytosociological relevés used in this study were sampled by different investigators in the field. Species trait dataset and pylogenetic tree both were obtained from public databases.

Data analysis

To analyse our data, we used the R statistical programming language (v.3.4.3, http://www.R-project.org). The R code for all analyses available here: https://github.com/jdivisek/NativeVsAlienTraits

For manuscripts utilizing custom algorithms or software that are central to the research but not yet described in published literature, software must be made available to editors/reviewers upon request. We strongly encourage code deposition in a community repository (e.g. GitHub). See the Nature Research [guidelines for submitting code & software](https://www.nature.com/documents/GuidelinesCodePublication.pdf) for further information.

## Data

*March 2018*

Policy information about [availability of data](http://www.nature.com/authors/policies/availability.html#data)

All manuscripts must include a [data availability statement](http://www.nature.com/authors/policies/data/data-availability-statements-data-citations.pdf). This statement should provide the following information, where applicable:

- Accession codes, unique identifiers, or web links for publicly available datasets
- A list of figures that have associated raw data
- A description of any restrictions on data availability

Phytosociological relevés used in this study can be obtained from the Czech National Phytosociological Database (GIVD code [EU-CZ-001; http://www.sci.muni.cz/](http://www.sci.muni.cz/)

botany/vegsci/dbase.php?lang=en) upon request. A list of species occurring in each habitat type is available in Supplementary Data. Species traits can be obtained

from the LEDA database https://uol.de/en/landeco/research/leda/. Phylogenetic tree used for phylogenetic corrections is available here: https:// dx.doi.org/10.6084/m9.figshare.c.3305040.v1

# Field-specific reporting

nature research | reporting summary

Please select the best fit for your research. If you are not sure, read the appropriate sections before making your selection.

Life sciences Behavioural & social sciences

For a reference copy of the document with all sections, see nature.com/authors/policies/ReportingSummary-flat.pdf

# Life sciences

## Study design

All studies must disclose on these points even when the disclosure is negative. Sample size

Our dataset consists of 24,935 vegetation plots (phytosociological relevés) obtained from the Czech National Phytosociological Database. This dataset is considered comprehensive across the range of the study system. Based on this dataset, a list of 1,438 native, 261 naturalized non- invasive and 50 invasive species occuring in the six broad vegetation types of Central Europe was compiled and further analysed.

Data exclusions

From the dataset of 24,935 vegetation plots belonging to the six considered habitats, we excluded all non-flowering plants because their traits (especially seed weight) are not directly comparable to those of flowering plants. We also removed juvenile trees from the samples of treeless habitats because species traits available in the databases were measured for fully grown individuals. All species whose invasion status was classified as "casual" (106) were not considered in the analyses because they occurred rarely in selected habitats.

Replication

This is an observational study with a big biodiversity data. Our tests were applied not only on intial dataset but also on i) the dataset where unavailable species trait values were imputed, and ii) the dataset where phylogenetic signal in species traits was accounted for. All analyses yielded very similar results. We provide all R codes for the analysis, thus the study can be fully reproduced.

Randomization

This is an observational study and experimental randomization is not possible for such a big system. However, we employed randomization tests to test statistical significance of observed patterns. These tests are clearly described in the Methods.

Blinding

Blinding is not relevant to this study. Phytosociological relevés were sampled across whole area of the Czech Republic by different investigators, but without any connection among them.

## Materials & experimental systems

Policy information about [availability of materials](http://www.nature.com/authors/policies/availability.html#materials)

n/a Involved in the study


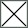

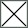

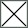

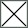

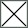


Unique materials Antibodies Eukaryotic cell lines Research animals

Human research participants

We require information from authors about some experimental systems used in the life sciences.

Here, indicate whether each material or system listed is relevant to your study.

If you are not sure if a material or system applies to your research, read the appropriate section below before selecting a response.

2

### Unique materials

Obtaining unique materials

*Describe any restrictions on the availability of unique materials OR confirm that all unique materials used are readily available from the authors or from standard commercial sources (and specify these sources).*

### Antibodies

Antibodies used

*Describe all antibodies used in the study; as applicable, provide supplier name, catalog number, clone name, and lot number.*

Validation

*Describe the validation of each primary antibody for the species and application, noting any validation statements on the manufacturer’s website, relevant citations, antibody profiles in online databases, or data provided in the manuscript.*

*March 2018*

### Eukaryotic cell lines

Policy information about [cell lines](http://www.nature.com/authors/policies/availability.html#celllines) Cell line source(s)

*State the source of each cell line used.*

Authentication

*Describe the authentication procedures for each cell line used OR declare that none of the cell lines used were authenticated.*

Mycoplasma contamination

*Confirm that all cell lines tested negative for mycoplasma contamination OR describe the results of the testing for mycoplasma contamination OR declare that the cell lines were not tested for mycoplasma contamination.*

nature research | reporting summary

Commonly misidentified lines

*Name any commonly misidentified cell lines used in the study and provide a rationale for their use.*

(See [ICLAC](http://iclac.org/databases/cross-contaminations/) register)

### Research animals

Policy information about [studies involving animals](http://www.nature.com/authors/policies/experimental.html); [ARRIVE guidelines](https://www.ncbi.nlm.nih.gov/pmc/articles/PMC2893951/) recommended for reporting animal research Animals/animal-derived materials

*For laboratory animals, report species, strain, sex and age OR for animals observed in or captured from the field, report species, sex and age where possible.*

### Human research participants

Policy information about [studies involving human research participants](http://www.nature.com/authors/policies/experimental.html) Population characteristics

*Describe the covariate-relevant population characteristics of the human research participants (e.g. age, gender, genotypic information, past and current diagnosis and treatment categories).*

Method-specific reporting

n/a Involved in the study


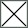

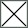

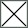


ChIP-seq

Flow cytometry

Magnetic resonance imaging

We also require information from authors about specific methods used in the life sciences. Here, indicate whether each method listed is relevant to your study.

If you are not sure if a method applies to your research, read the appropriate section below before selecting a response.

3

## ChIP-seq

### Data deposition

Confirm that both raw and final processed data have been deposited in a public database such as [GEO](https://www.ncbi.nlm.nih.gov/geo/). Confirm that you have deposited or provided access to graph files (e.g. BED files) for the called peaks.

Data access links

*For "Initial submission" or "Revised version" documents, provide reviewer access links. For your "Final submission" document, provide a link to the deposited data.*

*May remain private before publication.*

Files in database submission

*Provide a list of all files available in the database submission.*

Genome browser session

*Provide a link to an anonymized genome browser session for "Initial submission" and "Revised version" documents only, to enable peer review. Write "no longer applicable" for "Final submission" documents.*

(e.g. [UCSC](https://genome.ucsc.edu/index.html))

### Methodology

Replicates

*Describe the experimental replicates, specifying number, type and replicate agreement.*

Sequencing depth

*Describe the sequencing depth for each experiment, providing the total number of reads, uniquely mapped reads, length of reads and whether they were paired- or single-end.*

Antibodies

*Describe the antibodies used for the ChIP-seq experiments; as applicable, provide supplier name, catalog number, clone name, and lot number.*

Peak calling parameters

*Specify the command line program and parameters used for read mapping and peak calling, including the ChIP, control and index files used.*

Data quality

*Describe the methods used to ensure data quality in full detail, including how many peaks are at FDR 5% and above 5-fold enrichment.*

Software

*Describe the software used to collect and analyze the ChIP-seq data. For custom code that has been deposited into a community repository, provide accession details.*

## Flow Cytometry

*March 2018*

### Plots

Confirm that:

The axis labels state the marker and fluorochrome used (e.g. CD4-FITC).

The axis scales are clearly visible. Include numbers along axes only for bottom left plot of group (a 'group' is an analysis of identical markers).

4

All plots are contour plots with outliers or pseudocolor plots.

nature research | reporting summary

A numerical value for number of cells or percentage (with statistics) is provided.

### Methodology

Sample preparation

*Describe the sample preparation, detailing the biological source of the cells and any tissue processing steps used.*

Instrument

*Identify the instrument used for data collection, specifying make and model number.*

Software

*Describe the software used to collect and analyze the flow cytometry data. For custom code that has been deposited into a community repository, provide accession details.*

Cell population abundance

*Describe the abundance of the relevant cell populations within post-sort fractions, providing details on the purity of the samples and how it was determined.*

Gating strategy

*Describe the gating strategy used for all relevant experiments, specifying the preliminary FSC/SSC gates of the starting cell population, indicating where boundaries between "positive" and "negative" staining cell populations are defined.*

Tick this box to confirm that a figure exemplifying the gating strategy is provided in the Supplementary Information.

## Magnetic resonance imaging

### Experimental design

Design type

*Indicate task or resting state; event-related or block design.*

Design specifications

*Specify the number of blocks, trials or experimental units per session and/or subject, and specify the length of each trial or block (if trials are blocked) and interval between trials.*

Behavioral performance measures

*State number and/or type of variables recorded (e.g. correct button press, response time) and what statistics were used to establish that the subjects were performing the task as expected (e.g. mean, range, and/or standard deviation across subjects).*

### Acquisition

Imaging type(s)

*Specify: functional, structural, diffusion, perfusion.*

Field strength

*Specify in Tesla*

Sequence & imaging parameters

*Specify the pulse sequence type (gradient echo, spin echo, etc.), imaging type (EPI, spiral, etc.), field of view, matrix size, slice thickness, orientation and TE/TR/flip angle.*

Area of acquisition

*State whether a whole brain scan was used OR define the area of acquisition, describing how the region was determined.*

Diffusion MRI Used Not used

### Preprocessing

Preprocessing software

*Provide detail on software version and revision number and on specific parameters (model/functions, brain extraction, segmentation, smoothing kernel size, etc.).*

Normalization

*If data were normalized/standardized, describe the approach(es): specify linear or non-linear and define image types used for transformation OR indicate that data were not normalized and explain rationale for lack of normalization.*

Normalization template

*Describe the template used for normalization/transformation, specifying subject space or group standardized space (e.g. original Talairach, MNI305, ICBM152) OR indicate that the data were not normalized.*

Noise and artifact removal

*Describe your procedure(s) for artifact and structured noise removal, specifying motion parameters, tissue signals and physiological signals (heart rate, respiration).*

Volume censoring

*Define your software and/or method and criteria for volume censoring, and state the extent of such censoring.*

### Statistical modeling & inference

Model type and settings

*Specify type (mass univariate, multivariate, RSA, predictive, etc.) and describe essential details of the model at the first and second levels (e.g. fixed, random or mixed effects; drift or auto-correlation).*

*March 2018*

Effect(s) tested

*Define precise effect in terms of the task or stimulus conditions instead of psychological concepts and indicate whether ANOVA or factorial designs were used.*

Specify type of analysis: Whole brain ROI-based Both

5

Statistic type for inference

*Specify voxel-wise or cluster-wise and report all relevant parameters for cluster-wise methods.*

(See [Eklund et al. 2016](https://www.ncbi.nlm.nih.gov/pmc/articles/PMC4948312/))

nature research | reporting summary

Correction

*Describe the type of correction and how it is obtained for multiple comparisons (e.g. FWE, FDR, permutation or Monte Carlo).*

### Models & analysis

n/a Involved in the study

Functional and/or effective connectivity Graph analysis

Multivariate modeling or predictive analysis

Functional and/or effective connectivity

*Report the measures of dependence used and the model details (e.g. Pearson correlation, partial correlation, mutual information).*

Graph analysis

*Report the dependent variable and connectivity measure, specifying weighted graph or binarized graph, subject- or group-level, and the global and/or node summaries used (e.g. clustering coefficient, efficiency, etc.).*

Multivariate modeling and predictive analysis

*Specify independent variables, features extraction and dimension reduction, model, training and evaluation metrics.*

# Behavioural & social sciences

## Study design

All studies must disclose on these points even when the disclosure is negative. Study description

*Briefly describe the study type including whether data are quantitative, qualitative, or mixed-methods (e.g. qualitative cross-sectional, quantitative experimental, mixed-methods case study).*

Research sample

*State the research sample (e.g. Harvard university undergraduates, villagers in rural India) and provide relevant demographic information (e.g. age, sex) and indicate whether the sample is representative. Provide a rationale for the study sample chosen. For studies involving existing datasets, please describe the dataset and source.*

Sampling strategy

*Describe the sampling procedure (e.g. random, snowball, stratified, convenience). Describe the statistical methods that were used to predetermine sample size OR if no sample-size calculation was performed, describe how sample sizes were chosen and provide a rationale for why these sample sizes are sufficient. For qualitative data, please indicate whether data saturation was considered, and what criteria were used to decide that no further sampling was needed.*

Data collection

*Provide details about the data collection procedure, including the instruments or devices used to record the data (e.g. pen and paper, computer, eye tracker, video or audio equipment) whether anyone was present besides the participant(s) and the researcher, and whether the researcher was blind to experimental condition and/or the study hypothesis during data collection.*

Timing

*Indicate the start and stop dates of data collection. If there is a gap between collection periods, state the dates for each sample cohort.*

Data exclusions

*If no data were excluded from the analyses, state so OR if data were excluded, provide the exact number of exclusions and the rationale behind them, indicating whether exclusion criteria were pre-established.*

Non-participation

*State how many participants dropped out/declined participation and the reason(s) given OR provide response rate OR state that no participants dropped out/declined participation.*

Randomization

*If participants were not allocated into experimental groups, state so OR describe how participants were allocated to groups, and if allocation was not random, describe how covariates were controlled.*


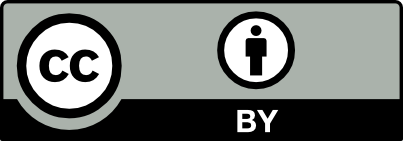
This checklist template is licensed under a Creative Commons Attribution 4.0 International License, which permits use, sharing, adaptation, distribution and reproduction in any medium or format, as long as you give appropriate credit to the original author(s) and the source, provide a link to the Creative Commons license, and indicate if changes were made. The images or other third party material in this article are included in the article's Creative Commons license, unless indicated otherwise in a credit line to the material. If material is not included in the article's Creative Commons license and your intended use is not permitted by statutory regulation or exceeds the permitted use, you will need to obtain permission directly from the copyright holder. To view a copy of this license, visit <http://creativecommons.org/licenses/by/4.0/>

*March 2018*
